# Monitoring the Stability of Perfluorocarbon Nanoemulsions by Cryo-TEM Image Analysis and Dynamic Light Scattering

**DOI:** 10.1371/journal.pone.0130674

**Published:** 2015-06-22

**Authors:** Christoph Grapentin, Sabine Barnert, Rolf Schubert

**Affiliations:** Department of Pharmaceutical Technology and Biopharmacy, Albert Ludwig University Freiburg i. Br., Freiburg im Breisgau, Germany; RMIT University, AUSTRALIA

## Abstract

Perfluorocarbon nanoemulsions (PFC-NE) are disperse systems consisting of nanoscale liquid perfluorocarbon droplets stabilized by an emulsifier, usually phospholipids. Perfluorocarbons are chemically inert and non-toxic substances that are exhaled after *in vivo* administration. The manufacture of PFC-NE can be done in large scales by means of high pressure homogenization or microfluidization. Originally investigated as oxygen carriers for cases of severe blood loss, their application nowadays is more focused on using them as marker agents in ^19^F Magnetic Resonance Imaging (^19^F MRI). ^19^F is scarce in organisms and thus PFC-NE are a promising tool for highly specific and non-invasive imaging of inflammation via ^19^F MRI. Neutrophils, monocytes and macrophages phagocytize PFC-NE and subsequently migrate to inflamed tissues. This technique has proven feasibility in numerous disease models in mice, rabbits and mini pigs. The translation to clinical trials in human needs the development of a stable nanoemulsion whose droplet size is well characterized over a long storage time. Usually dynamic light scattering (DLS) is applied as the standard method for determining particle sizes in the nanometer range. Our study uses a second method, analysis of transmission electron microscopy images of cryo-fixed samples (Cryo-TEM), to evaluate stability of PFC-NE in comparison to DLS. Four nanoemulsions of different composition are observed for one year. The results indicate that DLS alone cannot reveal the changes in particle size, but can even mislead to a positive estimation of stability. The combination with Cryo-TEM images gives more insight in the particulate evolution, both techniques supporting one another. The study is one further step in the development of analytical tools for the evaluation of a clinically applicable perfluorooctylbromide nanoemulsion.

## Introduction

Perfluoroalkanes are molecules in which all hydrogen atoms of the corresponding hydrocarbons are replaced by fluorine. They exist in linear and cyclic form [1]. The C-F bond is the strongest single bond known in organic chemistry. Fluorine being the most electronegative of all elements, the element has a high electron density, thus stabilizing the C-C backbone in perfluorocarbons (PFCs) and protecting them against degradation [2]. No enzymatic system is known to digest PFCs and they are chemically inert [3]. Outstanding chemical characteristics of PFCs are the extreme hydrophobicity as well as lipophobicity. The latter property can be modified by introducing heteroatoms like other halogens, oxygen or nitrogen. Nevertheless, water, oil and PFCs are immiscible with each other. PFCs usually display a high density due to their content in fluorine atoms and an elevated capacity to dissolve gases [4]. Clark and Gollan first demonstrated in 1966 that mice, fixed in a glass filled with oxygenated PFC, can survive, “breathing” the liquid PFC [5]. It could also be shown that isolated organs can remain functionality when perfused with oxygenated PFC [6].

Based on these findings, the first PFC emulsions for intravenous administration were developed. The major challenge in developing a PFC emulsion for parenteral use was that stable emulsions could only be obtained with PFCs that show a long biological half-life (several weeks), a feature that is not acceptable for clinical trials in human. For *in vivo* use, organ retention or biological half-life are decisive parameters. Excretion of PFCs happens by exhalation, determining parameters are the molecular weight of the PFC as well as its vapor pressure. The molecular weights range of applicable PFCs has been established between 460 and 520, which leaves just a narrow space for possibilities. PFCs are transported via blood fat to the lungs, this way increasing lipophilicity also enhances excretion [7]. Perfluorocarbon emulsions or nanoemulsions (PFC-NE) were first investigated as artificial oxygen carriers. Quite often blood is not available in the desired blood group and to a sufficient quantity, and blood transfusions also bear the risk of transmitting blood-borne diseases. Thus there is a strong need for a safe and well characterized PFC-NE for intravenous administration. Clinical trials have proven the efficacy of PFC-NE in transporting and delivering oxygen to tissues in human [8]. Though enormous efforts were made to develop a marketable formulation, until today no product has been approved for clinical use. The most promising candidate, Oxygent, was suspended from phase III clinical trials in humans in 2001 [9]. Oxygent contained a mixture of perfluorooctylbromide (PFOB) and its higher homologue perfluorodecylbromide (PFDB) and was overall well tolerated [10]. A clinical side-effect observed, leading to moderate influenza-like symptoms, was a macrophage activation caused by advanced uptake of the emulsion particles by cells of the reticuloendothelial system.

Being a side-effect in one application, the uptake of PFC-NE by monocytes and macrophages became the main factor in a second, nowadays widely investigated, field of application: ^19^F Magnetic Resonance Imaging (^19^F MRI). A new kind of MRI contrast agents was introduced in 2008 by Flögel et al. [11] who used PFC-NE for the visualization of, inter alia, myocardial infarction and stroke in mice via ^19^F MRI. Monocytes and macrophages play an important role in both the progression and resolution of inflammation [12]. Taking advantage of the phagocytosis of nanoscale contrast agent loaded particles by these innate immune cells and their subsequent migration to foci of inflammation, this technique has been used for the imaging of a huge variety of disease models connected with inflamed tissue. Probable applications range from such diverse pathological states as pulmonary inflammation [13], graft rejection [14], abscess formation [15], intratumoral inflammation [16] or neuroinflammation [17]. Usually a conventional ^1^H image is used for placing the ^19^F image into the anatomical location. Combined ^1^H/^19^F MRI features advantages over other imaging techniques like a high spatial resolution, excellent tissue contrast, the absence of harmful radiation or the avoidance of possibly toxic compounds like iron oxide or gadolinium, which are usually applied as contrast agents in ^1^H MRI [18–20].

Perfluorocarbons are ideal marker agents for ^19^F MRI because they are inert and non-toxic compounds with a high density of fluorine nuclei [4, 21]. ^19^F is the naturally occurring isotope of fluorine and scarce in organisms, thus obtained ^19^F signals are unambiguously stemming from applied PFC [22]. For parenteral administration, PFCs must be embedded into a biocompatible and biodegradable carrier system. Such nanoscale disperse systems contain a PFC core that is stabilized by a shell substance. Though for example PLGA [23, 24] or poloxamer [25] might be employed as well, the most often used emulsifiers are phospholipids [26]. For simple reasons, as phospholipids are well tolerated natural compounds approved for intravenous administration [27]. The formation of PFC-NE needs high energy input, which can be attained by means of high pressure homogenization or microfluidization [28, 29].

For ^19^F MRI, the preferred PFC is perfluoro-15-crown-5-ether (PFCE), its qualification being a facile spectrum that consists of one single peak stemming from 20 magnetically equivalent fluorine nuclei. However, a long biological half-life of several weeks renders it an unlikely candidate for clinical trials [30]. The most likely candidate for a clinical approach is perfluorooctylbromide (PFOB), also known as Perflubron, which is already characterized in the US Pharmacopoeia. PFOB shows a comparably short pulmonal elimination half-life of 2–3 days, depending on the dose, and due to its terminal bromine atom is slightly lipophilic and easily emulsified [7]. Though the NMR spectrum of PFOB is more difficult, leading to signal loss in MRI and thus requiring higher doses [31], the utility of PFOB-containing nanoemulsions in ^19^F imaging has already been proven in mice and mini pigs [32, 33].

A PFC-NE for intravenous administration must, considering a proposed market approval, fulfill several requirements. The nanoemulsion should carry a high loading of contrast agent that is excreted within an acceptable time frame to enable the repeated injection of small volume dosages. Sterility is a sine qua non for intravenous use and can be either achieved by filtration (0.22 μm filters) or heat sterilization under Pharmacopoeia standard conditions (121 °C, 2.05 bar, 15 min). Droplet size often hinders sterile filtration, emphasizing heat sterilization as the method of choice. The formulation should display stability when stored refrigerated at 4–8 °C. The use of phospholipids as emulsifying agents leads to the formation of PFC-free vesicles, i.e. liposomes, as a side product [34]. Liposomes are undesired, as they do not contain ^19^F MRI active material but due to their particulate character might overload the phagocytes and hence diminish the efficacy of the marker agent. Stability in case of PFC-NE should be defined as a constancy of nanoemulsion droplets in size as well as size distribution over time. The two principle mechanisms of degradation acting in a nanoemulsion are coalescence and Ostwald ripening. Both lead to irreversible droplet growth [35]. In coalescence, two nanoemulsion droplets attend each other, their surfaces form a contact phase and they finally merge, forming a bigger droplet. Repulsion of droplets by introducing a surface charge (zeta-potential) can diminish coalescence, which is supposed to be the primary mechanism of instability upon heat sterilization. The bigger droplet formed out of smaller ones has a respectively smaller total surface area than the smaller ones altogether. The disperse system aims at reducing the surface area to a lower value, a driving force of size instability. If the surface area becomes smaller, less emulsifier is needed to cover this area with a monomolecular layer. It is obvious, that in the case of phospholipids as emulsifiers, the formation of liposomes can, or must be, the consequence. In Ostwald ripening (also known as molecular diffusion) the droplets do not have to form a contact phase. In an emulsion with different droplet-sizes, molecules of the dispersed phase leave the smaller droplets due to curvature-induced high dissolution pressure and diffuse through the aqueous phase, then joining the larger droplets. This way, smaller droplets clear away while bigger droplets enlarge. This effect has its cause in the higher curvature of smaller particles leading to an increased capillary pressure following the Kelvin-effect. Ostwald ripening in a nanoemulsion can be slowed down by adding a small percentage of a higher homologue of the main dispersed component [36]. In case of using perfluorooctylbromide as the main PFC, its higher homologue perfluorodecylbromide, with an elevated tissue retention, might be added as a stabilizer [37, 38].

Size of particles of a disperse system in the nanoscale range is often monitored by means of dynamic light scattering (DLS). Though this technique holds some disadvantages when characterizing PFC-NE, it is widely used for this purpose. DLS measures the intensity of light scattered by moving particles, the amount of scattered light depending on the particles´ sizes. By doubling the radius, the scattered light multiplies sixfold, overestimating bigger sized particles. Furthermore, DLS is not capable of distinguishing between different particle types, i.e. buffer filled liposomes or PFC-filled nanoemulsion droplets. In preparations of PFC-NE, liposomes regularly occur with a size of 40–60 nm, which is smaller than the majority of PFC-filled nanoemulsions [39]. Advantages of DLS are fast and easy to perform measurements while using affordable standard equipment. DLS gives an averaged hydrodynamic size (d_Z_) and a size distribution (polydispersity index, PI) of particles in the sample. Using image analysis of e.g. Cryo-TEM, images it is possible to distinguish between liposomes and nanoemulsion droplets. Each particle type can be evaluated individually and number-weighted average diameters can be calculated [40]. However, this technique is a lot more laborious than DLS, it needs special expensive equipment and a skilled operator.

Scope of this work is the evaluation of stability of four perfluorocarbon nanoemulsions with varying composition by means of analysis of Cryo-TEM images compared to data obtained by dynamic light scattering. The impacts of total perfluorocarbon amount and the addition of a stabilizing second perfluorocarbon are considered as well as advantages and limits of the applied analytic tools. The obtained results are supposed to give impulses for aspects to consider when developing perfluorocarbon nanoemulsion for use as a marker agent in ^19^F MRI.

## Materials and Methods

### Materials

Purified soy phospholipid was a commercially available mixture (Lipoid S75, Lipoid, Ludwigshafen, Germany) containing ~70% phosphatidylcholine. Na_2_HPO_4_, NaH_2_PO_4_ and glycerol were from Carl Roth (Karlsruhe, Germany) and of analytical grade. Perfluorooctylbromide (purity 99%) and perfluorodecylbromide (purity 98%) were from ABCR (Karlsruhe, Germany).

The applied buffer contained 10 mM phosphate (7mM Na_2_HPO_4_, 3 mM NaH_2_PO_4_) and was isotonized with glycerol (2.5% w/w), the pH was adjusted to 7.2. For all preparation or analysis steps buffer was filtered and degased.

### Preparation of Nanoemulsions

Nanomulsions contained the same portion of phospholipid, 2.4% w/w, and either 23 or 63% w/w of PFOB or a mixture of PFOB and PFDB, buffer is added to 100%. To facilitate preparation recognition, samples are indexed as depicted in Table 1.

**Table 1:** Nanoemulsion composition and indices.

Index	Perfluorooctylbromide content (% w/w)	Perfluorodecylbromide content (% w/w)
**23**	23	0
**20/3**	20	3
**63**	63	0
**60/3**	60	3

The phospholipid was dispersed in 10 mM phosphate buffer, the PFC was added dropwise (either PFOB alone or a dissolution of PFDB in PFOB). A crude emulsion was formed by high shear mixing using a Miccra D9 mixer (tool DS-8/P) at 15,000 rpm for three minutes. The resulting crude emulsion was further processed on a Microfluidizer M110P (Microfluidics, Westwood, USA), equipped with a F20Y 75μ interaction chamber, for 10 cycles at 1000 bar process pressure, the preparation being cooled with ice water. All nanoemulsions were aliquoted in glass vials, sealed and underwent heat sterilization under standard conditions (121°C, 2.05 bar, 15 min). Aliquots of each nanoemulsion were stored at 4°C for 50 weeks. At distinct timepoints samples for analysis were taken.

### Dynamic light scattering (DLS)

The mean intensity-weighted hydrodynamic diameter was determined by dynamic light scattering (DLS) on a zetasizer nano (Malvern Instruments, Malvern, UK) directly after preparation of nanoemulsions, after heat sterilization and at distinct timepoints of storage. Prior to measurements the nanoemulsions were diluted 1:200 (v/v) with 0.22 μm filtered sample buffer. The measurements were performed at 25°C and at a scattering angle of 173°. Particle size is displayed as an averaged hydrodynamic diameter, d_z_. The width of the particle size distribution is expressed by the polydispersity index (PI). All measurements were performed in triplicates. Values are given as mean value of three measurements.

### Zeta potential measurements

The zeta potential was measured at 25°C by laser doppler anemometry using a zetasizer nano (Malvern Instruments, Malvern, UK). Samples were diluted 1:200 (v/v) with 0.22 μm filtered sample buffer. The zeta potential was determined in three measurements each consisting of ten subruns, data is given as the mean value.

### Cryo-Transmission electron microscopy and image analysis

Nanoemulsions were diluted with sample buffer to minimize droplet overlays and therefore facilitate following size measurements, samples of the same total PFC content were diluted in the same ratio to achieve comparability. Approximately 5 μl diluted dispersion were applied on a 400 mesh Quantifoil S7/2 holey carbon film on copper grids (Quantifoil Micro Tools GmbH, Jena, Germany). Excess liquid was removed from the grid with filter paper. The sample was then immediately shock-frozen by injecting it into liquid ethane. These sample preparation steps were done in a climate-controlled room using a CryoBox 340719 (Carl Zeiss, Oberkochen, Germany). The subsequent fixation of the grid on the sample rod (626-DH, Gatan, Warrendale, USA) and transfer of the rod into the transmission electron microscope (Leo 912 Ω-mega, Carl Zeiss, Oberkochen, Germany) were done under a nitrogen atmosphere at a temperature of 90 K (-183°C). The instrument was operated at 120 kV and pictures with a 6,300- to 12,500- magnification were taken from 12–21 different positions of the grid to include all particles of the sample (Camera: Proscan HSC 2, Oxford Instruments, Abingdon, USA) [41]. For each probe three grids were prepared and all images were used for further analysis unless particles appeared non-spherical due to deformation, an unfrequently occurring effect caused by a too thin water film e.g.

Nanoemulsion droplets can easily be distinguished from liposomes in Cryo-TEM images. Due to different light refraction properties buffer filled liposomes appear as light vesicles whereas perfluorocarbon filled nanoemulsion droplets appear dark. All nanoemulsion droplets detectable were measured, leading to count rates between 125 and 1758 droplets depending on the sample (total amount of PFC, storage time). The diameter of the particles was determined using iTEM 5.0 (Build 1054) software (soft imaging System GmbH, Münster, Germany). To proof accuracy of size measurements via image analysis three droplets of different sizes were measured independently fifty times each. Mean size, standard deviation (SD) and coefficient of variation (COV) were calculated. For a droplet of mean size 406.9 nm the SD of fifty measurements is 2.11 nm and COV is 0.52%. For a droplet of 156.5 nm mean size the SD is 2.82 nm and COV 1.80%. For a smaller droplet of mean size 94.9 nm the SD is calculated as 2.13 nm or 2.24%. This results indicate higher variations in size determination the smaller droplets become, still variation is in an acceptable range. The number weighted mean diameter with standard deviation, the median diameter and percentiles were calculated. Cumulative curves were drawn after classifying size distribution data in histograms with 20 nm increments, box plot diagrams were drawn from raw data.

## Results and Discussion

Regardless of addition of perfluorodecylbromide, nanoemulsions containing the same total amount of perfluorocarbon(s) show a similar average hydrodynamic size and size distribution when measured directly after preparation (Table 2). Nanoemulsions containing 63% w/w PFC are bigger sized than those containing 23% w/w due to a larger interfacial area at the same quantity of stabilizing phospholipid. After heat sterilization, an intense increase in average size is observed for all preparations. A stabilizing effect of perfluorodecylbromide is evident, as the preparations 20/3 and 60/3 show a diminished size increase and a narrower size distribution when compared to 23 and 63. Zeta potential is in a good range for all heat sterilized preparations that are subsequently stored.

**Table 2:** Initial dynamic light scattering analysis of nanoemulsions.

	freshly prepared		heat sterilized		
sample	d_Z_ [nm]	PI	d_Z_ [nm]	PI	ζ [mV]
**23**	129.4	0.135	191.2	0.257	-31.3
**20/3**	131.7	0.145	162.0	0.187	-32.3
**63**	186.0	0.060	235.7	0.128	-36.3
**60/3**	187.5	0.054	227.5	0.066	-34.1

d_z_ is the intensity-weighted hydrodynamic diameter as determined with dynamic light scattering. PI is the polydispersity index. ζ is the Zeta-Potential. Nanoemulsions carrying the same portion of PFC show a comparable size and size distribution when measured directly after preparation. Size and size distribution change during heat sterilization where those nanoemulsions without perfluorodecylbromide (PFDB) (23 and 63) grow more than their homologues with PFDB (20/3 and 60/3).

**Fig 1:**
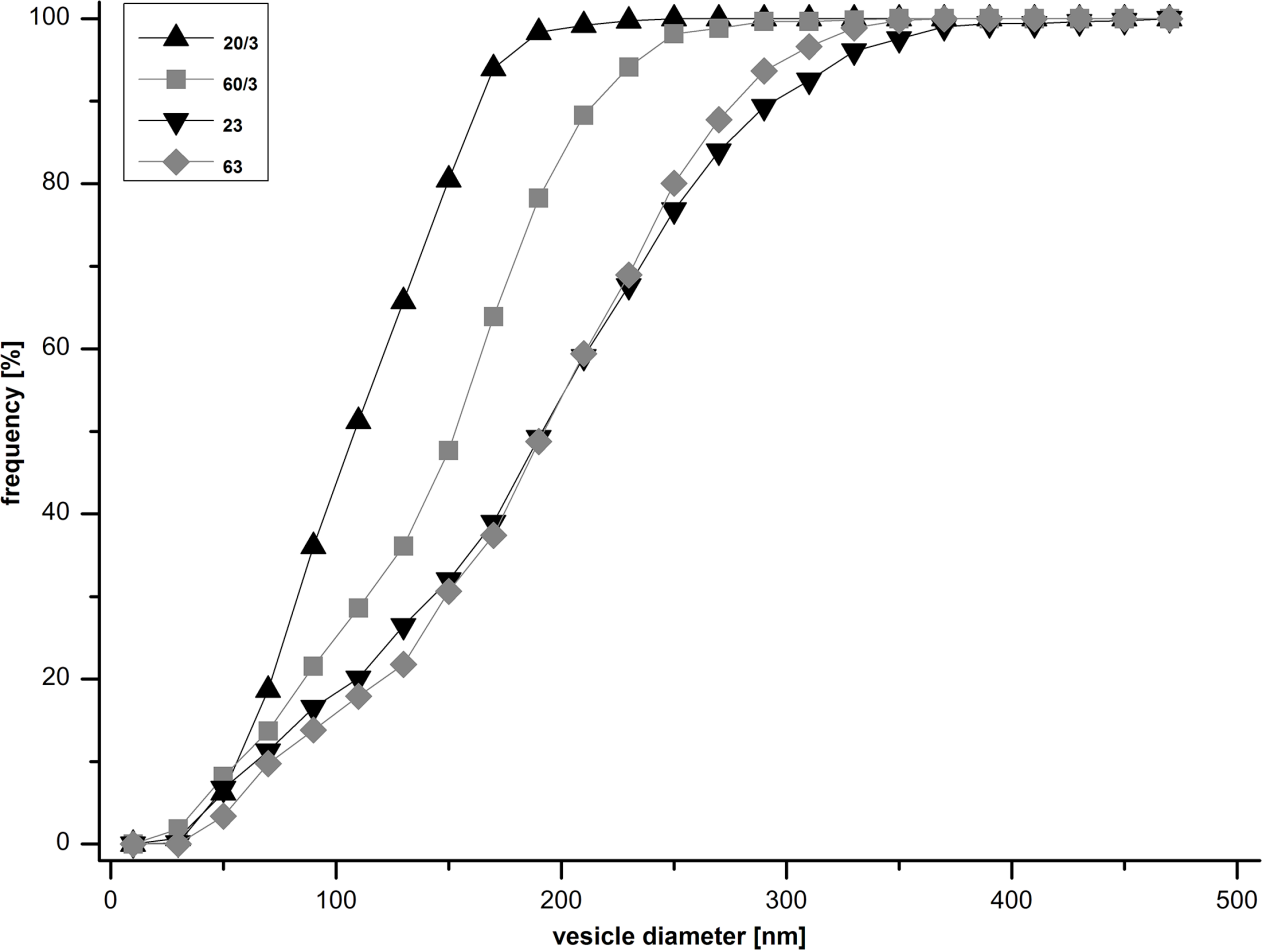
Frequency of nanoemulsion droplet sizes in heat sterilized preparations. Data obtained from Cryo-TEM image analysis. Nanoemulsions without added perfluorodecylbromide (23 and 63) show a broader size distribution that is shifted to bigger droplets as compared to nanoemulsions containing 3% perfluorodecylbromide (20/3 and 60/3). This indicates a stabilizing effect of perfluorodecylbromide in heat sterilization.

Size analysis of nanoemulsion droplets in Cryo-TEM images show an almost overlaying distribution curve for heat sterilized samples without added perfluoro-decylbromide (Fig 1). Nanoemulsions containing 3% PFDB show a size distribution shifted to smaller droplets, NE 20/3 naturally being the one with most small particles.

**Fig 2:**
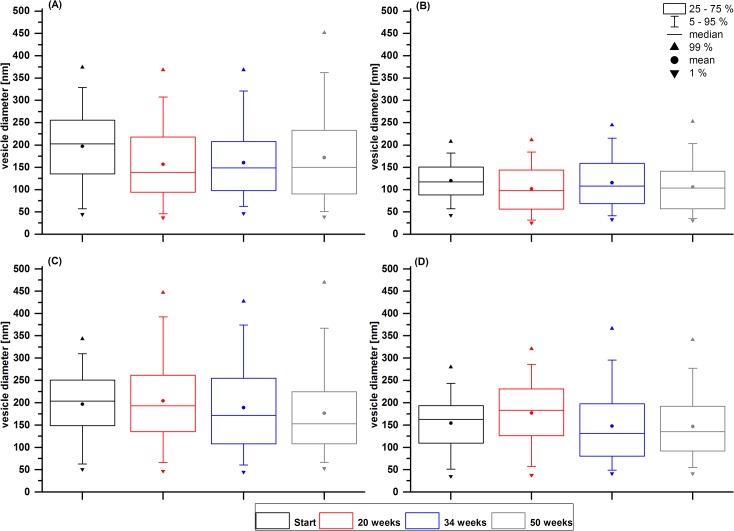
Box plot diagrams revealing droplet size distribution of nanoemulsions. Box plots show the development of droplet sizes over storage time for four nanoemulsions. Boxes represent droplet sizes with a probability between 25 and 75%, the line ▬ inside the box is for the median size. Mean diameter is indicated with a bullet ● and whiskers are for a probability of 5 (┴) or 95 (┬) % respectively. An arrow pointing downwards (▼) indicates the 1% value whereas an arrow pointing upwards (▲) represents the 99% probability of particle diameter. (A) is the nanoemulsion containing 23% perfluorooctylbromide (23), (B) the nanoemulsion with 20% perfluorooctylbromide and 3% perfluorodecylbromide (20/3). (C) represents the nanoemulsion with 63% perfluorooctylbromide (63) and (D) the one with 60% perfluorooctylbromide and 3% perfluorodecylbromide (60/3). Nanoemulsions lacking perfluorodecylbromide (A and C) show a broader size distribution and bigger mean size than their comparative nanoemulsions with stabilizing perfluorodecylbromide (B and D).

To determine stability after 20, 34 and 50 weeks, samples of stored nanoemulsions are evaluated in the same manner. Fig 2 shows box plot diagrams obtained from Cryo-TEM images of all four preparations whereas in Table 3 corresponding Cryo-TEM data are given and contrasted with DLS data.

**Table 3:** DLS and Cryo-TEM data of stored nanoemulsions.

	DLS data			Cryo-TEM data				
Sample	Timepoint	d_z_ [nm]	PI	d_n_ ± SD [nm]	median [nm]	d_P90_ [nm]	d_P95_ [nm]	d_P99_ [nm]
23	start	191.2	0.257	197.3 ± 83.9	202.8	305.6	329.1	374.2
	20 weeks	180.7	0.284	157.1 ± 85.0	138.6	277.9	307.5	368.4
	34 weeks	168.6	0.304	160.6 ± 77.8	148.9	268.4	321.3	368.3
	50 weeks	164.4	0.403	172.3 ± 98.8	149.9	315.6	362.0	451.6
20/3	start	162.0	0.187	120.1 ± 40.4	117.5	172.4	182.2	207.7
	20 weeks	169.6	0.175	102.0 ± 51.7	97.7	171.9	184.1	211.6
	34 weeks	165.3	0.164	115.6 ± 55.2	107.7	195.9	215.5	244.9
	50 weeks	172.1	0.194	105.8 ± 54.3	103.3	178.5	203.2	252.6
63	start	235.7	0.128	196.7 ± 73.3	203.7	289.9	309.5	342.8
	20 weeks	233.9	0.184	204.4 ± 97.2	193.1	330.7	392.7	446.9
	34 weeks	227.0	0.214	188.9 ± 100.1	171.4	331.1	374.2	427.1
	50 weeks	231.6	0.244	176.6 ± 93.2	152.8	306.6	367.4	469.8
60/3	start	227.5	0.066	154.2 ± 58.6	162.6	227.2	242.9	280.1
	20 weeks	236.1	0.086	177.3 ± 71.0	183.1	268.4	286.0	320.8
	34 weeks	220.4	0.085	147.8 ± 83.6	131.2	272.3	295.8	366.3
	50 weeks	227.9	0.098	147.0 ± 70.4	135.2	245.3	277.5	340.9

d_z_ is the intensity-weighted hydrodynamic diameter as determined with dynamic light scattering. PI is the polydispersity index. d_n_ is the number-weighted average diameter of nanoemulsion droplets from Cryo-TEM image analysis (= mean diameter in Fig 2). Median is the number-weighted size dividing the population of measured droplets in two halves. d_P90_, d_P95_ and d_P99_ are percentiles of droplet sizes, e.g. d_P90_ gives the number weighted size (diameter) of 90% of droplets being smaller and 10% bigger than this size.

**Fig 3:**
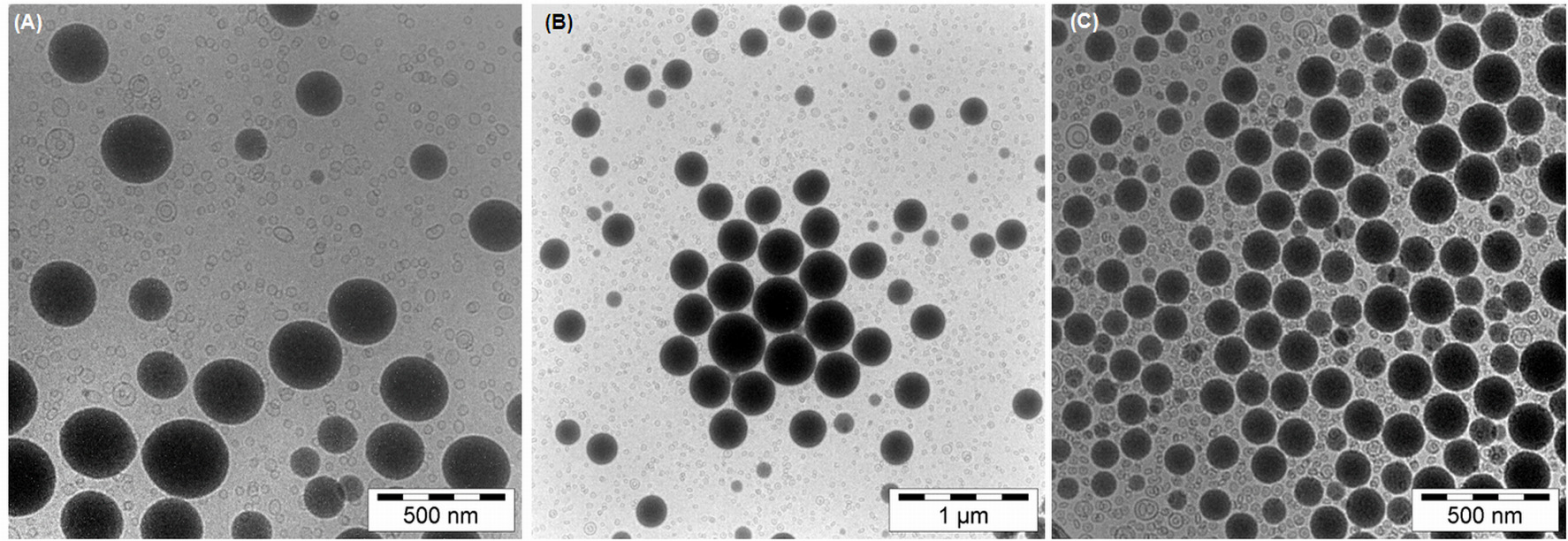
Sample Cryo-TEM images of perfluorocarbon-nanoemulsions. (A) Image of a nanoemulsion containing 23% w/w perfluorooctylbromide, after heat sterilization. Nanoemulsion droplets appear as dark vesicles, liposomes are visible to a hugh number as smaller, light vesicles. Scale bar is 500 nm. (B) shows the same nanoemulsion after 50 weeks storage at 4°C. Though the scale bar is 1 μm in this image, increased size of droplets can easily be observed. The number of liposomes seems to be stepped up. In (C) a nanoemulsion containing 20% perfluorooctylbromide and 3% perfluorodecylbromide after heat sterilization is depicted. Though a huge number of liposomes can be detected, more and smaller nanoemulsion droplets are visible than compared to image (A). All samples were diluted in the same ratio with sample buffer.

For the nanoemulsion 23, dynamic light scattering indicates a decreasing average size (d_z_) while PI worsens. The effect of Ostwald ripening in this preparation leads to shrinkage or disappearance of smaller sized droplets, while bigger sized droplets grow and small liposomes are formed by excess phospholipid, leading to a bimodal size distribution. Cryo-TEM data support this view, as the mean number-weighted size (d_n_) and median size diminish during storage time while the bigger sized droplets become constantly bigger, as can be seen in percentile values. Fig 3 shows sample Cryo-TEM images of NEs containing 23% PFC. It can be observed that nanoemulsion droplets become bigger and the number of liposomes increases during one year for nanoemulsion 23 (Fig 3(A) & 3(B)). Compared to nanoemulsion 20/3 in Fig 3(C), which shows smaller sized and more PFC containing vesicles after heat sterilization (compare with (A)). This NE 20/3, containing three percent of perfluorodecylbromide, is much more stable. DLS data reveal a slightly increasing hydrodynamic size at a slightly fluctuating, yet acceptable, PI below 0.2. Cryo-TEM data show a similarity to nanoemulsion 23 without added perfluorodecylbromide, as the mean and median size decrease and bigger sized droplets become more. Remarkably, this phenomenon is observed to a significantly smaller degree, Ostwald ripening is retarded.

NEs should contain a high portion of PFC to hold the volume of infused marker agent down. The NEs containing 63% (w/w) of PFC are at the outer edge of PFC that can be incorporated into a initially stable preparation. For nanoemulsion 63, DLS analysis shows an almost constant size, but the PI doubles during one year to a value above 0.2, indicating an inhomogeneous size distribution. Again, Cryo-TEM data reveal a decreasing mean and median size of PFC containing vesicles. The bigger sized droplets constantly become bigger. As for the NE containing 23% (w/w) PFC, the addition of 3% perfluorodecylbromide is capable of slowing down the process of droplet growth. The preparation 60/3 also shows a constant size, but in contrast to 63 remains a good PI value below 0.1. Cryo-TEM data support the view of a more stable nanoemulsion with droplets of a lower polydispersity.

Box plot diagrams in Fig 2 clearly illustrate the different droplet size distributions over time. NE 23 in Fig 2(A) starts with a wider size distribution and more bigger sized droplets when compared to NE 20/3 in Fig 2(B). Width of size distribution stays constant, but shifts to more smaller sized vesicles but some large ones for NE 23. Preparation 20/3 contains smaller sized droplets and remains more stable. The same effects can be observed for NE 63 and 60/3 in Fig 2(C) and 2(D).

## Conclusions

Perfluorocarbon nanoemulsions (PFC-NE) are a budding tool for imaging inflammatory foci via ^19^F Magnetic Resonance Imaging (^19^F MRI). After intravenous administration, unmodified crude nanoemulsions are phagocytized by immune cells which subsequently accumulate in inflamed tissue. Regarding an imaginable clinical proof of this concept, the need for the development of a well defined product, i.e. a highly concentrated, sterile nanoemulsion which stability is evaluated properly, emerges. Our results indicate that dynamic light scattering (DLS) alone is insufficient in determining the size of PFC-loaded droplets in such preparations. Due to mainly Ostwald ripening big droplets enlarge during storage, while smaller droplets become smaller or disappear completely. DLS only gives an averaged hydrodynamic diameter d_z_ as well as a size distribution (polydispersity index, PI) of all particles in the sample, not distinguishing liposomes from PFC-loaded nanoemulsion droplets. During storage time, d_z_ may stay constant or even decrease, a shift in particle size distribution is then indicated by the PI value. Occuring liposomes, which are usually smaller in size than nanoemulsion droplets, falsify the results by lowering the average size. By analysis of Cryo-TEM images one can distinguish between the two particle types, so the real size of nanoemulsion droplets can be measured unbiased, which gives a more detailed insight in droplet size distribution. Due to the different principle of size measurement, results of both techniques cannot be compared directly, but one method assists the other. For example, a constant average size at increasing PI as measured by DLS can be explained by a wide size distribution of a decreasing number of nanoemulsion droplets and a simultaneously existing high number of small liposomes as seen in Cryo-TEM images.

The most likely candidate for a clinical test of PFC-NE is perfluorooctylbromide (PFOB). NEs containing high loads of PFOB can be prepared by means of microfluidization. The addition of a small amount of a higher homologue, perfluorodecylbromide (PFDB), stabilizes the nanoemulsion against droplet growth during heat sterilization and storage at 4 °C. However, PFDB is not listed in a Pharmacopoeia and shows an elevated bio half-life, issues that considerably lower the probability of its use *in vivo*. Further research should focus on the preparation and analysis of PFOB nanoemulsions. More techniques for a quality control evaluating nanoemulsion droplet size and liposome content should be developed. Asymmetric flow field-flow fractionation might be capable of separating nanoemulsion droplets and liposomes by their size, in combination with static light scattering a deeper insight could be gained. This might be a promising future option in the development of a clinically applicable PFC-NE for *in vivo* ^19^F MRI, because only a well defined and characterized preparation can make it to clinical trials and approval.
